# Fire Resistance Performance of Steel–Polymer Prefabricated Composite Floors Using Standard Fire Tests

**DOI:** 10.3390/polym14071488

**Published:** 2022-04-06

**Authors:** Min Jae Park, Robel Wondimu Alemayehu, Young K. Ju

**Affiliations:** School of Civil, Environmental and Architectural Engineering, Korea University, Seoul 02841, Korea; alswo8739@korea.ac.kr (M.J.P.); robel@korea.ac.kr (R.W.A.)

**Keywords:** steel–polymer prefabricated composite floor, standard fire tests, fire resistance performance, stability, finite element analysis

## Abstract

In this study, the fire resistance performance of steel–polymer prefabricated composite floors, which have a sandwich-type structure, was assessed via standard fire tests and analyzed using finite element analysis. This form of analysis should consider two aspects, namely the thermal and structural fields, so as to simulate complicated material properties and large deformations. As previous studies have already conducted analysis in the thermal field, this study entailed only the structural analysis based on the temperature distributions obtained from the thermal analysis. The variables of the specimens were the thicknesses of the top and bottom steel plates and polymers. According to the analysis results, the top steel plate thickness had no impact on the stability ratings, a criterion for fire resistance performance, whereas the bottom steel plate showed a linear correlation with the stability rating. An equation for the stability rating of composite floors was proposed, and an equation for fire resistance performance was devised based on the insulation ratings, which were obtained from the thermal analysis results.

## 1. Introduction

Fire resistance, which can mitigate damage to life and property during building fires, is a significant capacity of structural members in buildings [1]. Fire resistance performance is evaluated using three criteria, namely integrity, insulation, and stability [2,3,4,5]. The integrity criterion examines the occurrence of flames, and the insulation criterion examines the temperature changes of unexposed surfaces under fire conditions. The stability criterion examines the deformation of structural members under fire conditions. For columns and beams that do not require compartments between the rooms, the evaluation of fire resistance performance needs only consider the stability criterion [6,7]. For floors and walls that require compartments between the rooms, the three criteria must be simultaneously considered for the evaluation [8,9,10]. Compartments are important in a building because they prevent the internal spread of fire and help the structural members in the compartments to remain stable under fire conditions. In particular, the fire resistance of floor members should be assessed to prevent large flexural deformations, which can cause the failure of interior materials and compartments. Without an assessment of the fire resistance performance and suitability of floor members, life and property, as well as the stability of buildings, would be endangered. Thus, several studies have been conducted on the fire behavior or fire resistance performance of various floor members [11,12,13,14,15]. Frangi et al. investigated the fire behavior of timber slabs made of hollow core elements using a reduced cross-section method, and considered the temperature-dependent charring phases of timber [11]. Frangi et al. proposed a fire design for timber–concrete composite slabs with screwed connections by considering the stress under tension and compression, and the strength of the screwed connection [12]. Romero et al. presented fire design methods for slim floor structures in thermal fields [13]. Frangi et al. and Menis et al. used experimental and numerical approaches to study the fire resistance of unprotected cross-laminated timber floor panels and proposed the failure time of floors by using simplified design methods [14,15].

**Figure 1:**
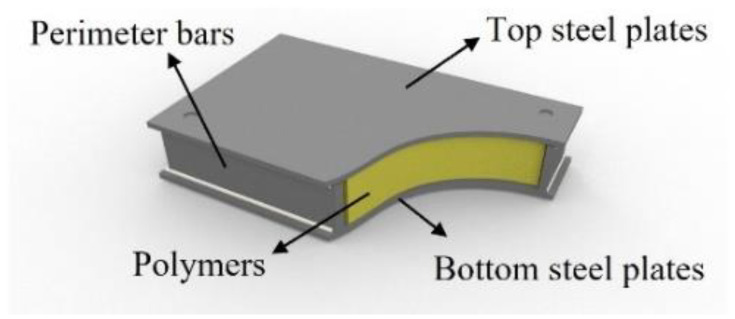
Steel–polymer prefabricated composite floors.

Recently, steel–polymer composite floors, which have a steel–polymer–steel sandwich-type section (Figure 1), have been developed for application in steel structures as prefabricated floor members to reduce the construction period and improve construction efficiency [16,17,18,19,20,21,22,23,24]. The thickness of steel–polymer prefabricated composite floors typically ranges from 25 mm to 80 mm. Composite deck slabs, which are general floor systems in steel structures, have a thickness of 100 mm to 250 mm. Despite their shallow thickness, these composite floors show an equivalent flexural capacity to that of composite deck slabs [16]. In addition, the vibration performance of composite floors has been investigated, with the results indicating that they could be installed in residential buildings given the energy absorption of the polymers [17]. Considering the shear deformation of polymers between steel plates, a new approach was proposed for estimating floor vibration performance with improved accuracy [18]. Experimental and numerical studies were conducted to investigate the fire behavior of steel–polymer prefabricated composite floors [19,20,21,22,23,24]. First, small-scale furnace tests were conducted to investigate the composite floor fire resistance, using an energy-based time equivalent approach [19]. Additionally, numerical studies were performed using finite element analysis. An analytical model of composite floors in the thermal field, considering the interfacial issues between steel and polymers, was proposed to predict the temperature distribution at elevated temperatures [20]. Using the proposed analytical model, verified by test results, a fire design equation in the thermal fields of composite floors was suggested [21]. Moreover, the insulation design procedure and worked examples under fire conditions for composite floor circular steel spacers were assessed using finite element analysis based on standard fire tests (known as full-scale furnace tests) [22]. Finite element models, considering the phase changes of polymers at elevated temperatures, were proposed to investigate the fire resistance performance of composite floors for the structural field. They were verified by fire tests with loading [23,24]. However, the fire resistance performance of standard fire tests, which can be used for designing composite floors under fire conditions for applications in buildings, has not been investigated.

Therefore, in this study, the fire resistance performance of steel–polymer prefabricated composite floors using standard fire tests [2,3,4,5] with temperature distribution [21] and reliable finite element models [23,24] was investigated to provide structural engineers with a fire design guide for composite floors. The thickness of the top and bottom steel plates and polymers were used to investigate the effects of these thicknesses on fire resistance performance.

## 2. Modeling for Standard Fire Tests

### 2.1. Test Setup

**Figure 2:**
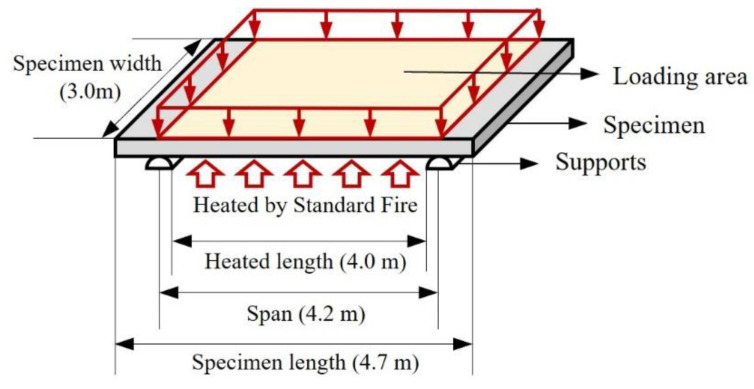
Standard fire test setup.

The standard fire tests for floor members developed by the International Organization for Standardization (ISO) [2,3] and the Korean Agency for Technology and Standards (KATS) [4,5] are illustrated in Figure 2. A specimen was installed on roller supports, and the bottom of the specimen between the supports was heated using a standard fire. The standard fire curve is expressed by Equation (1) in terms of time and initial room temperature. The size of the specimen was 4.7 m × 3.0 m. The span and heated lengths were 4.2 m and 4.0 m, respectively. A distributed load (1 kN/m^2^), which is half of the live load of an office area, was applied to the top of the specimen, as shown by the loading area in Figure 2 (yellow). The load was applied, and after 15 min the specimen was heated. The duration after heating when one of the criteria—integrity, insulation, and stability—was not satisfied constituted the fire resistance rating of the specimens.

(1) $$T=345\log_{10}\left(8t+1\right)+T_{0}$$

where *T* is the temperature of the standard fire, *t* is time, and *T*_0_ is the initial ambient temperature.

### 2.2. Test Specimens

**Figure 3:**
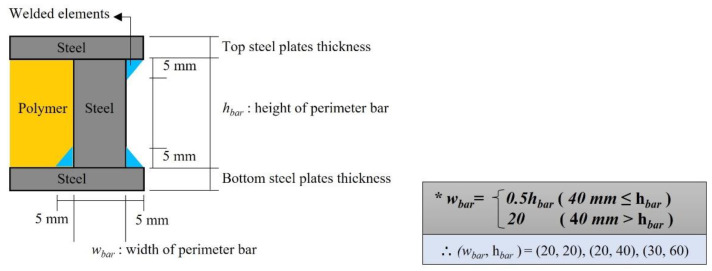
A detailed description of the perimeter bars.

Based on a previous study on the fire resistance performance of steel–polymer prefabricated composite floors in the thermal field, the specimen variables were the thickness of the top (*d_ts_*) and bottom (*d_bs_*) steel plates and polymers (*d_p_*). The total thickness (*d*) of the specimen was the sum of the thicknesses, *d_ts_*, *d_bs_*, and *d_p_*. A detailed illustration of the perimeter bar with respect to polymer thickness is presented in Figure 3. The perimeter bar width (*w_bar_*) was equal to half its height (*h_bar_*) for all specimens except for the specimen with a 20 mm polymer thickness, in which the width of the perimeter bar was equal to its height. The specimen list with variables is described in Table 1. During the manufacturing process, the bottom steel plate and perimeter bars were welded together at two points, whereas the top steel plates and perimeter bars were welded together at one point. The welded elements and their sizes are shown in Figure 3.

### 2.3. Failure Criteria

As aforementioned, there are three criteria for floor members in standard fire tests. As the steel–polymer prefabricated composite floors did not exhibit cracks under fire, the integrity criterion for the composite floors was not considered. In the standard fire test [2,3,4,5], the average and maximum temperature changes of the unheated surfaces were measured to evaluate the insulation criterion. The average temperature changes should not exceed 140 °C, and the maximum temperature changes should not exceed 180 °C. In the case of the stability criterion, the deformation and deformation rates of unheated surfaces should be evaluated. Equations (2) (deformation) and (3) (deformation rate) describe the stability criterion of floor members under fire. The criteria were proposed by Ryan and Bender [25] based on the numerous experimental results and slightly modified by ISO [2]. The deformation or the deformation rate of the specimen may not exceed these criteria, because that would imply that the specimen has lost its structural capacity. However, the deformation rate criterion should not be considered before the deformation exceeds *L_s_*/30.

(2) $$D=L_{s}^{2}/400d$$

(3) $$dD/dt=L_{s}^{2}/9000d,$$

where *D* is the deformation criterion, *L_s_* is the span of the specimen, *d* is the thickness of the specimen, and *dD/dt* is the deformation rate criterion.

## 3. Finite Element Analysis

### 3.1. Analysis Plan

**Figure 4:**
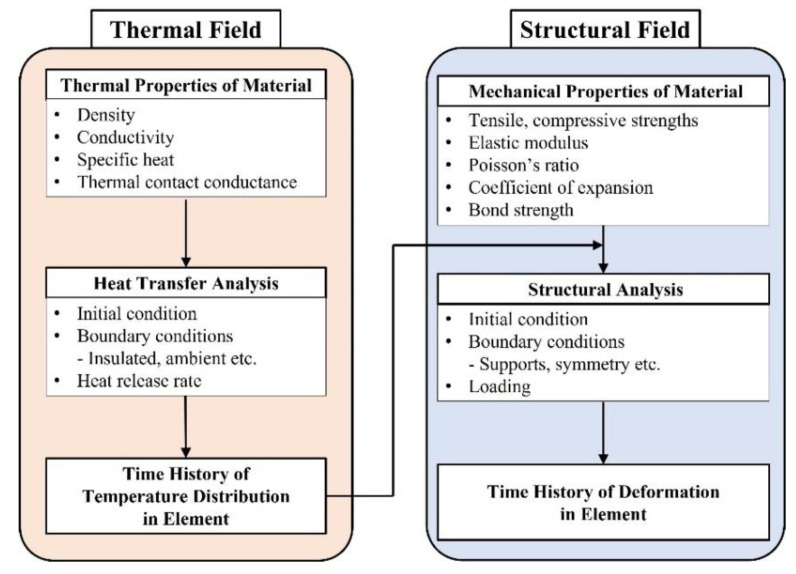
A specific analysis procedure.

Finite element analysis with the verified model using ABAQUS/CAE 2020 was conducted to evaluate the fire resistance performance of the steel–polymer prefabricated composite floors using a standard fire test. To simulate complicated material properties and large deformations, a heat transfer analysis is required to obtain the temperature distribution, followed by a structural analysis to obtain the deformation results [26,27]. A specific analysis procedure for composite floors under fire is shown in Figure 4. In the thermal field, to obtain the time dependent temperature distribution, heat transfer analysis was conducted based on thermal properties, such as density, conductivity, specific heat, and thermal contact conductance between the contact surfaces. Using this temperature distribution, a structural analysis was conducted based on the various mechanical and interfacial properties between the contact surfaces to obtain the deformation of the element over time. Because the heat transfer analysis for obtaining the temperature distribution has been conducted in a previous study [21], in this study, a structural analysis of the temperature distribution was conducted with a verified finite element model [20,24]. For this analysis, an eight-node linear brick was selected to comprise the mesh; based on previous studies, the maximum size of the mesh was set as 10 mm [16,20,21,22,23,24]. Owing to the shallow thickness of the composite floor, the polymer mesh thickness, which was expected to be largely deformed, was 5 mm.

### 3.2. Thermal Properties

The polymer used in this study was a type of amorphous polyurethane, formed by reacting diisocyanate containing phosphorous with a polyol mixture. The molecular formula of the polymer is OHRO[RP(=O)]OROH. The phase-change temperatures of the polymers in this study were inconsistent in specific ranges because the amorphous polyurethanes had irregular and numerous polymer chains. Through several material property tests, the phase-change temperature ranges of the amorphous polyurethane were measured, as presented in Table 2 [21,23,24]. Furthermore, the selected phase-change temperatures are also presented in Table 2, because the fire resistance performance was evaluated under disadvantageous conditions, such as the lowest phase-change temperature and the lowest strength.

### 3.3. Mechanical Properties

Mechanical properties that are temperature-dependent, such as strength, elastic modulus, and Poisson’s ratio, in addition to the thermal expansion coefficients of steel and polymers, are required to simulate the fire behavior of the steel–polymer prefabricated composite floors using finite element analysis. The temperature-dependent mechanical properties of steel were obtained from Eurocode 3 [28]. In the case of polymers, temperature-dependent validated mechanical properties and phase changes [23,24] were applied, as presented in Table 3. The thermal expansion coefficients were obtained using Equation (4), based on the verified results [24].

(4) $$\alpha_{cp}\left(T\right)=\left\{\begin{array}{cc} \alpha_{p} & \left(T<420\;{}^{\circ}C\right)\\ \frac{E_{p}\left(T\right)\alpha_{p}\left\{V_{p}\left(T\right)-V_{pg}\left(T\right)\right\}+E_{p}\left(T\right)\left\{\lambda /E_{p}\left(T\right)+\alpha_{g}\left(T\right)\right\}V_{pg}\left(T\right)}{E_{p}\left(T\right)\left\{V_{p}\left(T\right)-V_{pg}\left(T\right)\right\}+E_{p}\left(T\right)V_{pg}\left(T\right)} & \left(420\;{}^{\circ}C\leq T\right) \end{array}\right.$$

Where *α_cp_*(*T*) is the thermal expansion coefficient of the phase-change polymers depending on the temperature, *T* is the temperature, *α_p_* is the thermal expansion coefficient of the polymers at the ambient temperature (0.000203), *E_p_*(*T*) is the elastic modulus of phase-change polymers depending on the temperature, *V_p_*(*T*) is the volume of phase-change polymers depending on the temperature, *V_pg_*(*T*) is the volume of gasified polymers depending on the temperature, *λ* is the volumetric expansion by gasification of the polymers (50), and *α_g_*(*T*) is the thermal expansion coefficient of the gas depending on the temperature.

### 3.4. Interfacial Properties

The three cases in the finite element analysis should be defined. The first and second cases are related to friction at the interface of materials during loading. The first case relates to the contact between the supports and the specimen. In this case, it was the contact between steel and steel. The second case is the contact between the steel and polymers in the specimens. The friction coefficients should be defined to simulate the interfacial properties along with the normal and tangential components. For the normal component, a hard contact was selected; this implies that there is no deformation along the normal axis. For the tangential component, the friction coefficients for the different polymer phase-change states were selected based on previous studies (Table 4) [16,23,24].

**Figure 5:**
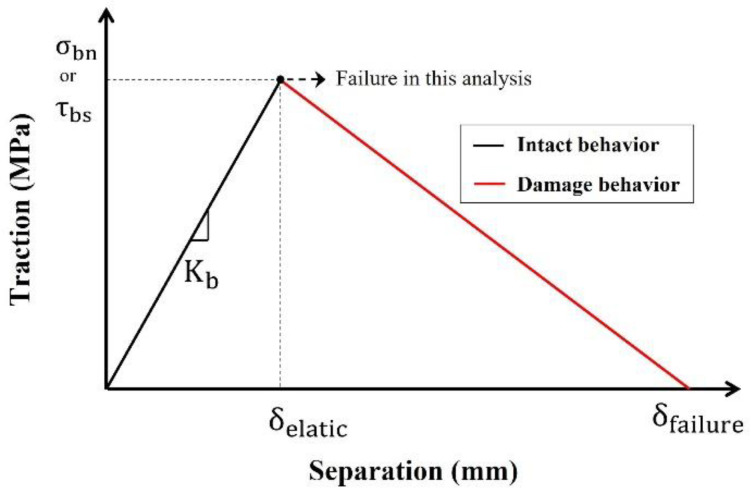
Cohesive zone model (traction-separation constitutive relation).

The third case is related to the bond between the two materials (steel and polymers) and its detachment at the contact surfaces caused by the loads. A cohesive zone model, known as the traction-separation constitutive relation [29], has in previous studies simulated the bond between steel and polymers in composite floors [16,23,24]. The cohesive zone model is illustrated in Figure 5. The intact behavior showed that traction and separation had a linear relation with stiffness (*K_b_*) before the traction approached maximum values for normal bond strength (*σ_bn_*) or shear bond strength (*τ_bs_*). The normal and shear bond strengths between steel and polymers were obtained from previous studies (Table 5) [23,24]. The stiffness in the cohesive zone model was assumed as the elastic modulus according to previous studies [16,24]. The minimum strengths were selected for this study, and the damage (red line in Figure 5) was ignored to consider conservative conditions.

The quadratic traction criterion was selected in this study to model the debonding between steel and polymers, as shown in Equation (5):

(5) $$\sqrt{{\left(\frac{\sigma_{bn}}{\sigma_{bn,\max}}\right)}^{2}+{\left(\frac{\tau_{bs,1}}{\tau_{bs,\max}}\right)}^{2}+{\left(\frac{\tau_{bs,2}}{\tau_{bs,\max}}\right)}^{2}}\leq 1,$$

where *σ_bn_* is the normal bond strength, *σ_bn,max_* is the maximum normal bond strength, *τ_bs_* is the shear bond strength, and *τ_bs,max_* is the maximum shear bond strength.

Based on previous studies, the stiffness was assumed to be the temperature-dependent elastic modulus, described in Table 3. The reduction ratio of the maximum normal and shear bond strengths at elevated temperatures was assumed to be equal to the reduction ratio of the tensile strength at elevated temperatures, also described in Table 3. To avoid the penetration at the contact between the steel and polymers, the dense meshes for the polymers were selected, as mentioned in Section 3.1.

### 3.5. Validation

**Figure 6:**
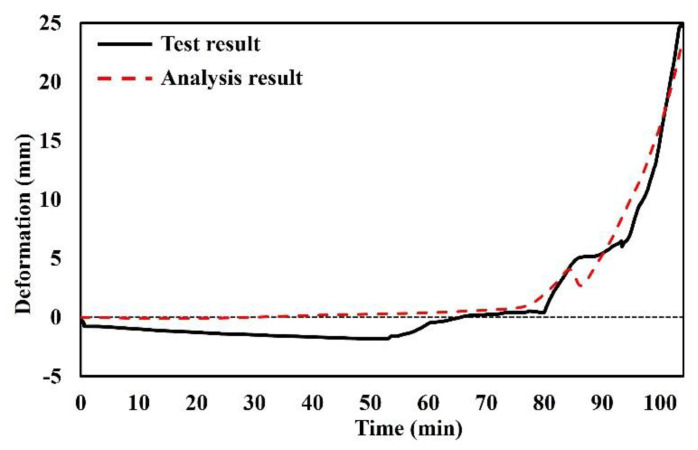
Comparison between analysis and test results.

To obtain the reliability of the proposed finite element model, the analysis result that simulated the fire test was compared to the test result [24]. The thickness of the polymer was 60 mm, and the top and bottom steel plates were 5 mm, respectively. The size of the specimen was 450 mm × 450 mm, and the span was 400 mm. A distributed load (1 kN/m^2^) was applied to the top of the specimen. The gasification temperature of the specimen was selected as 500 °C. The comparison between analysis and test results is shown in Figure 6. From the comparison, the validation of the proposed finite element model was obtained.

## 4. Fire Resistance Performance

### 4.1. Analysis Results

**Figure 7:**
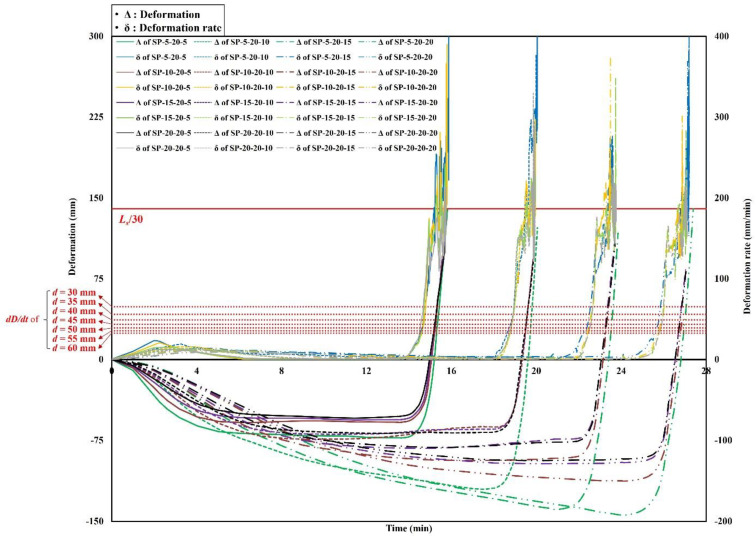
Time history of deformations and deformation rates of *d_p_* = 20 mm.

**Figure 8:**
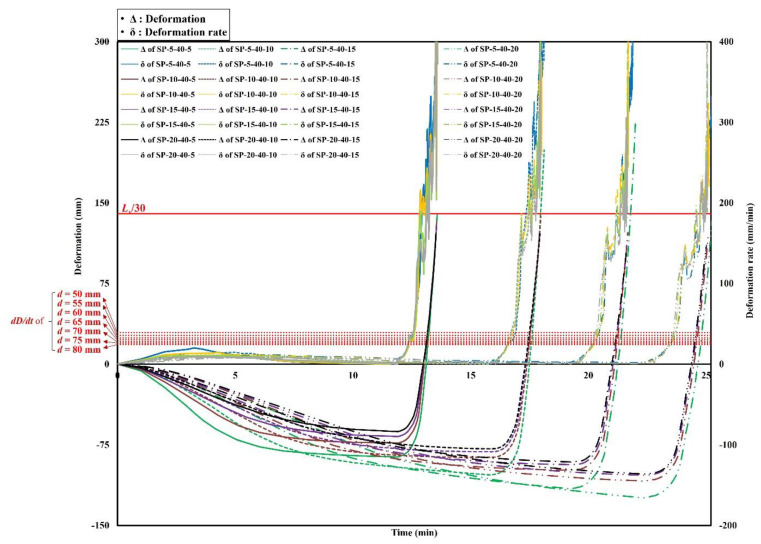
Time history of deformations and deformation rates of *d_p_* = 40 mm.

**Figure 9:**
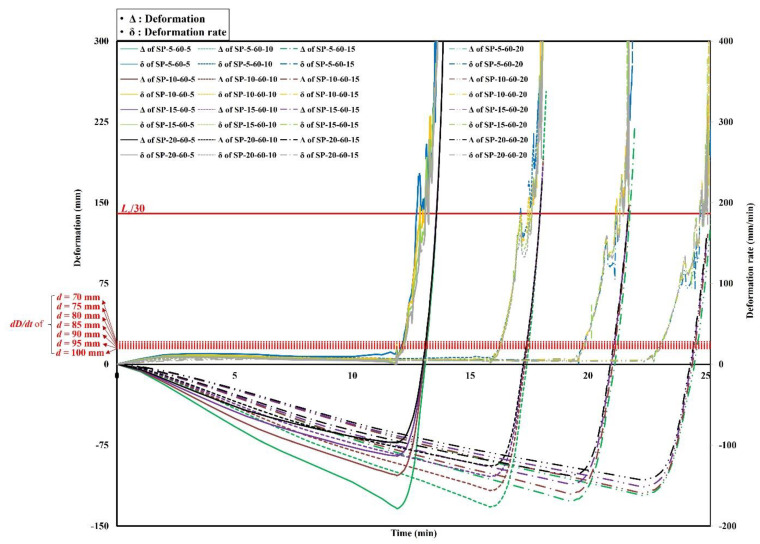
Time history of deformations and deformation rates of *d_p_* = 60 mm.

The analysis results of the deformations and deformation rates of the steel–polymer prefabricated composite floors under standard fire tests are shown in Figure 7, Figure 8 and Figure 9. The results of the specimens with a polymer thickness of 20 mm, 40 mm and 60 mm are shown in Figure 7, Figure 8 and Figure 9, respectively. Deformation of the center of the top steel plate was measured based on standard fire tests. In the early stages, the specimens deformed downwards before the polymers were gasified. After gasification, the polymers rapidly expanded. Owing to the expansion, the bottom and top steel plates receded. All specimens exceeded the deformation rate criterion before exceeding the deformation criterion. However, to evaluate the deformation rates, the deformations need to first exceed *L_s_*/30. Therefore, the stability ratings of the specimens were determined when the deformations exceeded *L_s_*/30. Specimens with thinner polymers experienced drastic downward deformations that were maintained prior to polymer gasification. Before the expansion, the deformation rates were relatively low. Due to the drastic expansion, however, the deformations rates rapidly increased.

### 4.2. Discussions

Based on the analysis results, the stability ratings for the different thicknesses of the top and bottom steel plates and polymers are listed in Table 6. Based on these results, two significant points were identified. First, the thickness of the top steel plates did not affect the stability ratings of specimens with constant thickness of the bottom steel plate and polymers. Second, the stability ratings and various thicknesses of the bottom steel plates with a constant thickness of the top steel plate and polymers exhibited a nearly linear correlation. This implies that the thickness of the top steel plate did not contribute to the structural performance of the steel–polymer prefabricated composite floor under fire. However, the bottom steel plate thickness contributed linearly to the stability of the composite floor under fire. This was because of the temperatures of the bottom and top steel plates. Because the bottom steel plate was exposed to high temperatures, the strength and stiffness were much lower than that of the top steel plate. Therefore, the effect of the thickness of the bottom steel plate was relatively greater than that of the top steel plate. Based on the correlations between the thickness and stability ratings, the equation for estimating the stability rating of steel–polymer prefabricated composite floors under standard fire tests can be written as Equation (6):

(6) $$S=\left\{\begin{array}{cc} 13-0.1d_{p}+0.8d_{bs} & \left(20\leq d_{p}<40\right)\\ 9+0.8d_{bs} & \left(40\leq d_{p}\leq 60\right) \end{array}\right.$$

where *S* is the stability rating, *d_p_* is the polymer thickness, and *d_bs_* is the thickness of the bottom steel plates.

An expression for the fire resistance performance of steel–polymer prefabricated composite floors under standard fire tests, using the insulation rating from a previous study (Equation (7)) [21], is proposed by Equation (8):

(7) $$I=\left\{\begin{array}{c} 1)d_{ts}>10\left\{\begin{array}{cc} 6.5d_{p}+0.4d_{s}-100 & \left(20\leq d_{p}<40\right)\\ 0.2d_{p}+0.4d_{s}+24 & \left(40\leq d_{p}\leq 60\right) \end{array}\right.\\ 2)d_{ts}\leq 10\left\{\begin{array}{cc} 8.7d_{p}+1.2d_{ts}+0.4d_{bs}-0.04d_{p}d_{ts}-154 & \left(20\leq d_{p}<40\right)\\ 0.5d_{p}+1.2d_{ts}+0.4d_{bs}-0.04d_{p}d_{ts}+21 & \left(40\leq d_{p}\leq 60\right) \end{array}\right. \end{array}\right.$$

(8) $$FR=Min\left[S,I\right],$$

where *I* is the insulation rating, *d_p_* is the thickness of the polymers, *d_ts_* is the thickness of the top steel plate, *d_s_* is the total thickness of the top and bottom steel plates, *d_bs_* is the bottom steel plate, and *FR* is the fire resistance rating.

## 5. Conclusions

In this study, the fire resistance performance of the steel–polymer prefabricated composite floor was investigated under standard fire tests using finite element analysis. A structural analysis was conducted to evaluate the fire resistance performance of the composite floor using the temperature distributions obtained from previous studies. The findings of this study may be summarized as follows:

(1) The top steel plate thickness showed negligible effects on the stability ratings of steel–polymer prefabricated composite floors under standard fire tests, whereas the bottom steel plate thickness exhibited a linear relationship with the stability ratings. In addition, thicker polymers resulted in lower stability ratings due to polymer expansion at high temperatures;
(2) An estimated stability rating was proposed for steel–polymer prefabricated composite floors under standard fire tests. It is expressed in terms of the thickness of the bottom steel plates (*d_bs_*) and polymers (*d_p_*) with specific ranges of 5 mm ≤ *d_ts_*, *d_bs_* ≤ 20 mm, and 20 mm ≤ *d_p_* ≤ 60 mm, as follows: $S=\left\{\begin{array}{cc} 13-0.1d_{p}+0.8d_{bs} & \left(20\leq d_{p}<40\right)\\ 9+0.8d_{bs} & \left(40\leq d_{p}\leq 60\right) \end{array}\right.$
(3) On the basis of previous studies, the fire resistance performance of steel–polymer prefabricated composite floors under standard fire tests was proposed using Equation (8), which is written in terms of the thicknesses of the top and bottom steel plates and polymers. The fire resistance performance was determined as the minimum value between the insulation and stability ratings.

## Figures and Tables

**Table 1:** Specimen list with variables (unit: mm).

Specimens	*d_ts_*	*d_p_*	*d_bs_*	*d*	*h_bar_*	*w_bar_*
SP-5-20-5	5	20	5	30	20	20
SP-10-20-5	10			35		
SP-15-20-5	15			40		
SP-20-20-5	20			45		
SP-5-20-10	5		10	35		
SP-10-20-10	10			40		
SP-15-20-10	15			45		
SP-20-20-10	20			50		
SP-5-20-15	5		15	40		
SP-10-20-15	10			45		
SP-15-20-15	15			50		
SP-20-20-15	20			55		
SP-5-20-20	5		20	45		
SP-10-20-20	10			50		
SP-15-20-20	15			55		
SP-20-20-20	20			60		
SP-5-40-5	5	40	5	50	40	20
SP-10-40-5	10			55		
SP-15-40-5	15			60		
SP-20-40-5	20			65		
SP-5-40-10	5		10	55		
SP-10-40-10	10			60		
SP-15-40-10	15			65		
SP-20-40-10	20			70		
SP-5-40-15	5		15	60		
SP-10-40-15	10			65		
SP-15-40-15	15			70		
SP-20-40-15	20			75		
SP-5-40-20	5		20	65		
SP-10-40-20	10			70		
SP-15-40-20	15			75		
SP-20-40-20	20			80		
SP-5-60-5	5	60	5	70	60	30
SP-10-60-5	10			75		
SP-15-60-5	15			80		
SP-20-60-5	20			85		
SP-5-60-10	5		10	75		
SP-10-60-10	10			80		
SP-15-60-10	15			85		
SP-20-60-10	20			90		
SP-5-60-15	5		15	80		
SP-10-60-15	10			85		
SP-15-60-15	15			90		
SP-20-60-15	20			95		
SP-5-60-20	5		20	85		
SP-10-60-20	10			90		
SP-15-60-20	15			95		
SP-20-60-20	20			100		

**Table 2:** Ranges of phase-change temperatures of the polymers [21,23,24].

Phase-Change Temperatures	Lower Bound (°C)	Upper Bound (°C)	Selected Temperatures (°C)
Glass transition (*T_g_*)	260	260	260
Softening (*T_s_*)	280	310	280
Melting (*T_m_*)	310	370	310
Charring (*T_c_*)	370	420	370
Gasification (*T_gas_*)	420	500	420

**Table 3:** Mechanical properties of the polymers depending on the temperatures [23,24].

Temperature (°C)	Strength (MPa)	Elastic Modulus (MPa)	Poisson’s Ratio
20	25.9	1050	0.33
100	0.76	13.2	0.33
200	0.38	9.72	0.33
260	0.20	8.78	0.33
420	0.04	0.04	0.00
1200	0.00	0.00	0.00

**Table 4:** Friction coefficients [16,23,24].

Steel and Steel	Steel and Polymers	
Friction Coefficient	Phase Changes of Polymers	Friction Coefficients
0.3	Solid state	0.3
	Liquefied state	0.1
	Charred state	0.1
	Gasified state	0.02

**Table 5:** Bond strengths [23,24].

	Normal Bond Strength (MPa)	Shear Bond Strength (MPa)
Maximum	8.94	3.9
Average	4.78	2.5
Minimum (selected)	1.08	0.8

**Table 6:** Stability ratings depending on the thickness of the top and bottom steel plates and polymers.

***d_p_* 20 mm**	*d_ts_* (mm)	5				10				15				20			
	*d_bs_* (mm)	5	10	15	20	5	10	15	20	5	10	15	20	5	10	15	20
	Stability (min)	15	20	23	27	15	20	23	27	15	20	23	27	15	20	23	27
***d_p_* 40 mm**	*d_ts_* (mm)	5				10				15				20			
	*d_bs_* (mm)	5	10	15	20	5	10	15	20	5	10	15	20	5	10	15	20
	Stability (min)	13	17	21	25	13	17	21	25	13	17	21	25	13	17	21	25
***d_p_* 60 mm**	*d_ts_* (mm)	5				10				15				20			
	*d_bs_* (mm)	5	10	15	20	5	10	15	20	5	10	15	20	5	10	15	20
	Stability (min)	13	17	21	25	13	17	21	25	13	17	21	25	13	17	21	25

## Data Availability

Not applicable.
